# Detection of Genetically Modified Maize in Processed Foods Sold Commercially in Iran by Qualitative PCR

**Published:** 2013

**Authors:** Maryam Rabiei, Mehrangiz Mehdizadeh, Hossein Rastegar, Hossein Vahidi, Mahmoud Alebouyeh

**Affiliations:** a *Food and Drug Laboratory Research Center, Food and Drug Organization, Ministry of Health and Medical Education, Tehran, Iran.*; b *Department of Molecular Biology, Food and Drug Control Reference Laboratory, Ministry of Health and Medical Education, Tehran, Iran.*; c *Department of Biotechnology, Faculty of Pharmacy, Shahid Beheshti University of Medical Sciences and Health Services, Tehran, Iran.*

**Keywords:** GMO, Maize, Detection, DNA extraction, Polymerase chain reaction.

## Abstract

Detection of genetically modified organisms (GMOs) in food is an important issue for all the subjects involved in food control and customer’s right. Due to the increasing number of GMOs imported to Iran during the past few years, it has become necessary to screen the products in order to determine the identity of the consumed daily foodstuffs. In this study, following the extraction of genomic DNA from processed foods sold commercially in Iran, qualitative PCR was performed to detect genetically modified maize. The recombinant DNA target sequences were detected with primers highly specific for each investigated transgene such as CaMV35s gene, Bt-11, MON810 and Bt-176 separately. Based on the gel electrophoresis results, Bt- 11 and MON810 events were detected in some maize samples, while, in none of them Bt- 176 modified gene was detected. For the first time, the results demonstrate the presence of genetically modified maize in Iranian food products, reinforcing the need for the development of labeling system and valid quantitative methods in routine analyses.

## Introduction

 Genetic modification has been an extremely valuable tool in plant genetic research. The genetically modified organism (GMO) contains an exogenous gene which is able to express a new protein that confers new traits, *i.e*. resistance to virus, insect, herbicide tolerance or improved nutritional value (1, 2). Many genetically modified crops have been approved worldwide since mid-1990s and the global area of these crops has expanded yearly since their commercialization. In 2011, 160 million hectares were cultivated with genetically modified crops mainly soybean, maize, cotton and canola (3,4). At the moment, several transgenes have been approved worldwide for cultivation and consumption as food and feed, while consumer›s increasing awareness regarding food safety has created the need for more stringent regulations to control raw materials, import, export and distribution of GMOs (7, 8). Therefore, the detection and identification of GMOs in food have become important issues for all the subjects involved in food control (9, 10). Qualitative testing may be used to discriminate between the authorized and unauthorized GM food to identify safe or potentially unsafe material (11, 12).

 Although several analytical methods have been proposed, GMO detection methods generally involve specific DNA sequence detection by means of PCR techniques, able to detect even small amounts of transgenes in raw materials and processed foods (13, 14). In this study, we examined the detection of recombinant DNA of genetically modified maize in several processed foodstuffs gathered from Iranian market.

## Experimental

*DNA extraction*

 All the samples were grounded and homogenized with an electric mill (IKA m20 WERKE). DNA extraction was carried out from 100 mg of ground and homogenized foodstuff based on the CTAB method (1, 15). This method includes the following steps: first, 300 μL of sterile deionized water was added to the sample and mixed. To the mixture, 500 μL of CTAB extraction buffer (20 g/L CTAB, 1.4 M NaCl, 0.1 M Tris-HCl, 20 mM Na_2_EDTA) was added and mixed. Then, 20 μL Proteinase K (20 mg/ mL) was added, mixed and incubated at 65°C for 90 min. Then, 20 μL of RNase A (10 mg/ mL) was added, mixed, incubated at 65°C for 5-10 min and centrifuged for 10 min at 16000 g. Afterwards, the supernatant was transferred to a new tube containing 500 μL chloroform, mixed for 30 sec and centrifuged for 10 min at 16000 was added, mixed and centrifuged for 10 min at 16000 g. Then, the supernatant was discarded and the pellets were dried and re-dissolved in 100 μL sterile deionized water.

*From reference material*

 Appropriate reference materials for positive and negative controls provide the basis for the validation of analytical procedures (15). The following commercially available Certified Reference Materials were used: GM-free maize powders (ERM-BF411a, ERM-BF412a, ERMBF413a), maize powder containing 5% of Bt-176 maize (ERM-BF411f), maize powder containing 5% of Bt-11 maize (ERM-BF412f), maize powder containing 5% of MON810 maize (ERM-BF413f). All CRMs were obtained from Joint Research Center, Institute for Reference Materials and Measurements (JRC-IRMM), Geel, Belgium.

*From processed food samples*

 In total 25 food samples including a variety of processed material, from relatively mild treated maize to highly processed products containing maize ingredients, for human consumption were gathered randomly from different Iranian supermarkets. The analyzed products were as follows: 4 corn flakes, 3 frozen maize, 5 corn puffs, 5 corn seeds, 4 canned corns, 2 corn chips and 2 pop corns.

*Evaluation of the concentration and the purity of the extracts*

 Following nucleic acid extraction, the concentration of DNA was measured by UV absorption at 260 nm using Biophotometer plus apparatus (Eppendorf), and DNA purity was evaluated on the basis of the UV absorption ratio at 260/280 nm. The 260/280 nm ratio of the extracted DNA of all the samples ranged from 1.7 to 2 (15).

*Oligonucleotide primers*

 Oligonucleotide primers were purchased as purified and desalted specimen from Eurofins. The primers were diluted to a final concentration of 10 μM with sterile double-distilled water and stored at - 20°C until use. The sequences of oligonucleotide primers are given in Table 1.

**Table 1:** Oligonucleotide primers used to detect species- specific or transgenic DNA sequences in food by polymerase chain reaction (PCR).

**Target**	**Sequence**	**Primer**	**Amplicon length (bp)**	**Ref**
Maize zein	CGC CAG AAA TCG TTT TTC AT	MZ for	139	13
	GGT GGT GTC CTT GCT TCC TA	MZ rev		
CaMV35s	GCT CCT ACA AAT GCC ATC A	35s-1	195	16
	GAT AGT GGG ATT GTG CGT CA	35s-2		
Bt-11	CTG GGA GGC CAA GGT ATC TAA T	Intron IVS2-2	189	16
	GCT GCT GTA GCT GGC CTA ATC T	PAT-B		
MON810	CAT TTC ATT TGG AGA GGA CAC G	P-E35S for	110	13
	GCA TTC AGA GAA ACG TGG CAG TA	MON810 rev		

*PCR conditions*

 DNA extraction was followed by qualitative PCR protocols using different set of primers. Conventional PCR for the detection of *zein*, CaMVp35s, Bt-11, MON810 and Bt176 genes were carried out using a Mastercycler gradient instrument (Eppendorf) in a final volume of 20 μL with the following reagent concentrations: PCR buffer 1x (CinnaGene), MgCl_2_ 2.5 mM (CinnaGene), dNTPs 0.2 mM each (CinnaGene), primers forward and reverse 0.5 μM each (Eurofins MWG Operon), Taq DNA polymerase 0.025 U/ μL (CinnaGene), Template genomic DNA 100 ng. In order to obtain reliable results, for each set of primers, positive, negative and no-template controls were used during the PCR reactions (16). Certified reference materials of GM-free maize flour (ERM-412a) as negative control and maize flour containing 5% of Bt-11 maize (ERM-412f), 5% of MON810 maize (ERM-413f) and 5% of Bt-176 maize (ERM-411f) were used as positive controls. No-template control of the master mix containing water instead of DNA was used to control the environmental contamination. Thermal cycler conditions were as follows: preincubation at 95°C for 4 min, 34 cycles consisting of dsDNA denaturation at 95°C for 50 sec, primer annealing for 45 sec at 52°C for *zein*, 54°C for CaMV35s, 59°C for Bt-11, 56°C for MON810 and 61°C for Bt 176 primers respectively; primer extension at 72°C for 50 sec; and final elongation at 72°C for 5 min. To find the best annealing temperature for each set of primers in our laboratory, we used PCR gradient programs. 

*Agarose gel electrophoresis*

 PCR products were analyzed using agarose gel electrophoresis. The gel was prepared with 1.5% agarose (Merck), in Tris Borate EDTA (TBE) 0.5x (Sigma) with 10 μg/mL of DNA Safe Stain (CinnaGene). The conditions were constant voltage at 120 V for 1 h in TBE 0.5 x buffer. To visualize the stained amplicons, Transilluminator Villber Lourmat® imaging system was employed.

## Results and Discussion

 DNA isolated by the CTAB method from the low processed food samples were of good quality which was checked by amplifying the endogenous *zein *gene, expecting to find a fragment of 139 bp. Agarose gel electrophoresis together with spectrophotometric results revealed DNA of high purity. On the other hand, it was hardly possible to obtain good quality DNA for PCR from highly processed food such as corn flakes and corn puffs.

 Since currently corn is the second major GM crop grown worldwide, the food samples containing maize were chosen to be analyzed as follows: one corn puff, two corn chips, one corn seed and one frozen corn (Table 2). 

**Table 2:** The results of 25 analyzed maize samples.^*^

**Product’s name**	**Number of samples** **analyzed**	Domestic**/Imported final product	**Result (GM/Non GM)**	**GM label** **(Present/Absent)**
1 Corn flakes	4	2 domestic, 2 imported	All non GM	Absent
2 Frozen maize	3	All domestic	1 GM, 2 non GM	Absent
3 Corn puffs	5	All domestic	1 GM, 4 non GM	Absent
4 Corn seeds	5	3 domestic, 2 imported	1 GM (domestic), 4 non GM	Absent
5 Canned corns	4	2 domestic, 2 imported	All non GM	Absent
6 Corn chips	2	All domestic	All GM	Absent
7 Pop corns	2	All domestic	All non GM	Absent

*All samples contain production license, **May be made of imported raw materials.

**Figure 1:**
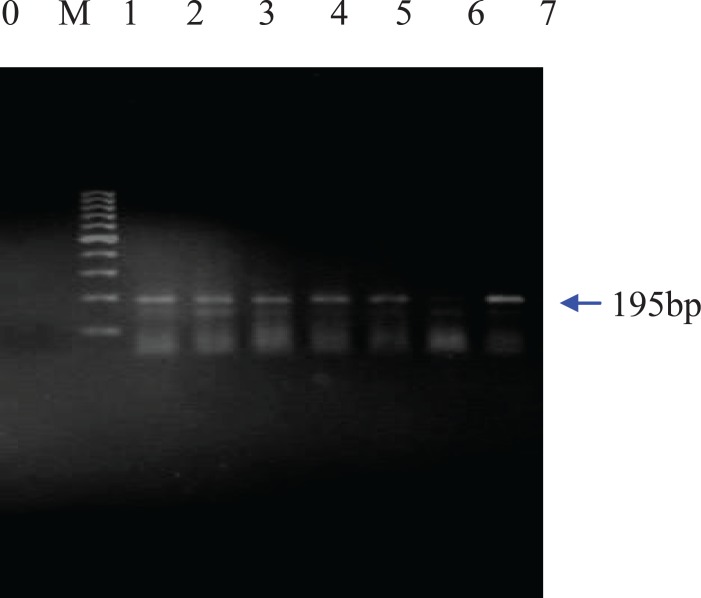
Agarose gel electrophoresis of PCR products from DNA of food samples containing CaMV35s**.**

 For this purpose, different PCR analyses were carried out using different primers such as *zein,* CaMV35s, Bt-11, Bt-176 and MON810. At first, plant-specific primers for the maize intrinsic *zein *gene were used to confirm the presence of amplifiable maize DNA extracted from the samples. Then, screening method based on the detection of the common regulatory sequence inserted in most GM products, the 35s promoter, was performed. Genetic control elements such as the *Cauliflower Mosaic* virus 35s promoter are present in around 95% of currently commercialized GMO plants in EU (17). Finally, GMO-specific primers for the selective detection and identification of the transgenic maize line Bt-11, Bt-176 and MON810 were used. According to the PCR products, 5 out of 25 foods showed positive results with CaMV35s primers confirming that they contain genetically modified genes (Figure 1).

Lane 1-5: DNA extracted from five food samples; Lane 7: DNA extracted from positive control (ERM-412f); M: DNA ladder (100bp).6: No Template Control; 0: Negative control.

**Figure 2:**
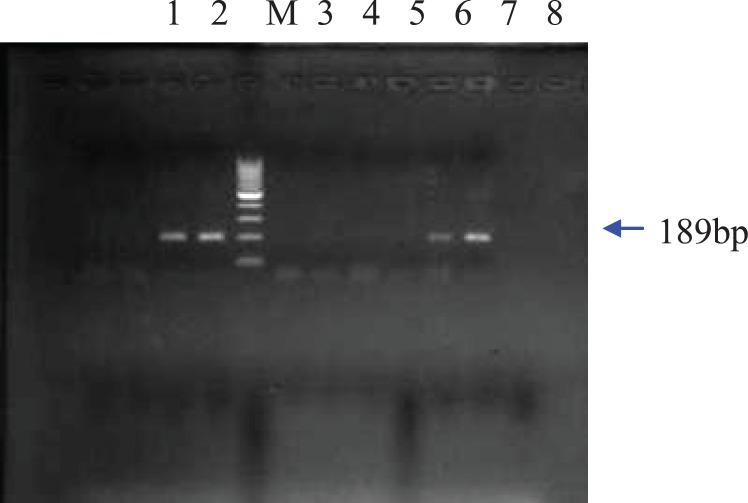
Agarose gel electrophoresis of PCR products from DNA of 3 food samples containing Bt-11.

 All the five CaMVp35s positive samples were chosen to be analyzed by PCR with specific primers of Bt-11, MON810 and Bt- 176. Based on the gel electrophoresis results, Bt-11 event was detected in the corn puff and the two corn chips (Figure 2). The two corn chips samples which were Bt- 11 positive also contained MON810 event. In none of the samples, Bt-176 modified gene was detected. Genetically modified maize such as Bt-11, MON810 and Bt-176 contain genes from the bacterium *Bacillus thuringiensis *so that the plant will produce insecticidal protein crystals active against certain lepidopteron insects such as European Corn Borer (ECB) (18). Maize event Bt-11 is designed to be both insect-resistant and herbicide-tolerant and has been approved by EU as food or feed. Maize event MON810 approved by EU for food and feed use was developed to be resistant to ECB and to provide a method to control yield losses without using conventional pesticides. Maize event Bt- 176 is also resistant to ECB. In addition, this maize line was co-transformed with a gene that confers tolerance to the herbicide glufosinate ammonium (1).

Lane 1, 2, 7: DNA extracted from CaMV35s positive food samples; Lane 8: DNA extracted from positive control (ERM-412f); M: DNA

ladder (100 bp); 5: Negative control; 6: No template control.

 As expected, a band of 139 bp for the *zein* gene was amplified for all extracted genomic DNAs of the maize food samples. Moreover, agarose gel electrophoresis of the PCR amplified products from five maize samples resolved a band of 195bp for the introduced gene of CaMV35s promoter (Figure 1). As CaMV35s gene is common in most of the currently commercialized GMO plant, the DNA extracts that showed positive results in the CaMV35s screening method, were further specifically analyzed. Figure 2 shows that the amplification product of 189 bp from event Bt-11 has been found in three samples. The agarose gel of the PCR products resulted from PCR using MON 810 primers revealed a band of 110 bp for this maize event in two samples which have shown positive results for Bt-11 event either. In none of the samples, Bt 176 event was detected. The gels in which all the positive, negative and notemplate controls showed appropriate results were interpreted.

## Conclusion

 Although genetically modified plants have some potential benefits, there exist safety concerns regarding the GMO consumption as food or feed (19). According to legislation in EU and several other countries, products containing GMO should be approved and labeled, which demand reliable and accurate methods to detect GMO in raw materials and food products (20, 21). In the present study, genetically modified DNA was detected in the processed food products which were gathered from Iranian market. DNA isolation from the samples was performed by the CTAB extraction method that has been widely applied in molecular genetics of plants (22). The obtained DNA from different food matrices were of appropriate quality for PCR amplification. Polymerase chain reactionbased (PCR-based) methods are very sensitive, reliable and frequently used in food analysis.

 According to the results, it was shown that the two samples which were different types of corn chips products from the same food company contained both MON810 and Bt-11 events. Similarly, in a previous study by Grenier *et al. *detecting modified maize in processed food sold commercially in Brazil, it was demonstrated that in the products which scored positive for the presence of genetically modified maize, two maize events such as MON810 and Bt-11 were detected in a single product like tortilla chips (23). The results of our study clearly demonstrate the presence of genetically modified maize in the Iranian food market, emphasizing the need for the development of quantitative PCR methods in routine analyses and implementing labeling systems for genetically modified food products.
